# Usefulness of Linear Mixed-Effects Models to Assess the Relationship between Objective and Subjective Internal Load in Team Sports

**DOI:** 10.3390/ijerph18020392

**Published:** 2021-01-06

**Authors:** Alice Iannaccone, Daniele Conte, Cristina Cortis, Andrea Fusco

**Affiliations:** 1 Department of Human Sciences, Society and Health, University of Cassino e Lazio Meridionale, Viale dell’Università, 03043 Cassino, Italy; alice.iannaccone@unicas.it (A.I.); c.cortis@unicas.it (C.C.); 2 Institute of Sport Science and Innovations, Lithuanian Sports University, Sporto g. 6, 44221 Kaunas, Lithuania; daniele.conte@lsu.lt

**Keywords:** team sports, statistical analysis, correlation, monitoring, RPE, heart rate, beach handball, training load, youth athletes

## Abstract

Internal load can be objectively measured by heart rate-based models, such as Edwards’ summated heart rate zones, or subjectively by session rating of perceived exertion. The relationship between internal loads assessed via heart rate-based models and session rating of perceived exertion is usually studied through simple correlations, although the Linear Mixed Model could represent a more appropriate statistical procedure to deal with intrasubject variability. This study aimed to compare conventional correlations and the Linear Mixed Model to assess the relationships between objective and subjective measures of internal load in team sports. Thirteen male youth beach handball players (15.9 ± 0.3 years) were monitored (14 training sessions; 7 official matches). Correlation coefficients were used to correlate the objective and subjective internal load. The Linear Mixed Model was used to model the relationship between objective and subjective measures of internal load data by considering each player individual response as random effect. Random intercepts were used and then random slopes were added. The likelihood-ratio test was used to compare statistical models. The correlation coefficient for the overall relationship between the objective and subjective internal data was very large (*r* = 0.74; ρ = 0.78). The Linear Mixed Model using both random slopes and random intercepts better explained (*p* < 0.001) the relationship between internal load measures. Researchers are encouraged to apply the Linear Mixed Models rather than correlation to analyze internal load relationships in team sports since it allows for the consideration of the individuality of players.

## 1. Introduction

Monitoring athletes’ workload is an essential process to understand the level of adaptation to a given training program and it is useful in minimizing the risk of nonfunctional overreaching [1,2]. The workload can be either external and internal, where the external load represents an objective measure of the work performed by the athlete (i.e., total distance covered in different speed zones and the number of sprints, accelerations, and decelerations), while the internal load represents the psychophysiological response of the athlete to a given training stimulus [3].

External load can be assessed by means of Global Positioning Systems (GPS), Inertial Movement Units (IMU) [3], accelerometers [3], and Local Positioning Systems (LPS) [4]. Although these systems are widely used in team sports such as basketball [5,6,7], handball [8], and beach handball [9], they present several limitations, such as high cost, the need of high technical expertise, and the risk of technical errors leading to a loss of data [10].

Internal load indicates the functional outcome of a given external load and can be used as an inexpensive way of monitoring athletes [11]. Internal load can be measured by means of objective methods such as heart rate (HR), blood lactate concentration, and oxygen uptake, and it is useful for improving performance and evaluating maladaptive responses to training programs [2,12,13]. HR is the most commonly adopted objective parameter used for monitoring internal load in team sports [1], with many HR-based models such as the Summated Heart Rate Zone (SHRZ) model [14]. Internal load can be also evaluated subjectively using questionnaires, such as the session Rating of Perceived Exertion (sRPE), which is among the most commonly used in team sports [3,15]. The advantages of using the sRPE include its ease of use and interpretation and its ability to provide information not only on the physiological responses to the prescribed load but also the psychological responses [16]. Moreover, the sRPE represents a valid tool for monitoring internal load when HR monitoring is not possible [17]. To use the sRPE as an alternative to HR-based methods, it is warranted to assess its validity, which represents the extent to which method results are associated with those of other accepted methods that measure the same parameter [18]. For this purpose, simple correlations have been previously adopted as main statistical tests to assess the concurrent validity of objective and subjective methods for monitoring the internal load, proving that the sRPE method is a valid, alternative tool to HR-based methods. However, when using simple correlation analyses, the within-subject variability it is not considered [19].

One way to overcome this limitation and improve the statistical analysis is the use of Linear Mixed Model (LMM) [20], which involves a generalization of linear regression but with both fixed and random effects. Fixed effects are analogous to the linear predictor from a standard linear regression, while the random effects are not directly estimated but are summarized according to their estimated variances and covariances. This structure gives additional flexibility to the statistical model, making it possible to model the random intercept and random slope as independent, correlated, or independent with equal variances [21]. In addition, LMMs make it possible to handle missing data instead of withdrawing subjects from the analysis. However, to the best of our knowledge, no previous study has applied LMMs to analyze the relationship between the subjective and objective methods used for monitoring the process of internal load in team sports. Therefore, the present study aims to (1) assess the correlation between objective and subjective internal load measures in team sports and (2) investigate these relationships by taking into account the individuality of players by means of LMMs.

## 2. Materials and Methods

### 2.1. Participants

Thirteen youth male players were recruited from the Lithuanian Under 17 beach handball team and volunteered to participate to this study. All players were novice to beach handball, but they had regularly trained for at least 5 years in indoor handball. Prior to the beginning of the study, all players, their parents, and the coaching staff were informed about the study aim, procedures, potential risks, and benefits associated with participation, and informed consent was obtained from participants’ parents. The study was approved by the Institutional Review Board of the Department of Human Sciences, Society and Health of the University of Cassino and Lazio Meridionale (approval number: 3R1B.2019.05.06) according to principles outlined in the Declaration of Helsinki.

### 2.2. Experimental Design

Players’ internal loads were monitored across 2 training camps (14 training sessions) and during the Young Age Category 17 European Beach Handball tournament held in Stare Jablønki (Poland) from the 27 to 30 June 2019 where players were involved in 7 matches. Data were excluded from the analysis if players did not complete the entire session due to possible injuries. In total, data were collected across 21 sessions, resulting in 192 (136 trainings and 57 matches) individual values. The average temperature of the training sessions and matches was 20.5 ± 3.5 °C and the relative humidity was 65 ± 17.7%. To provide ecological conditions during the training sessions, the team’s coaching staff freely planned their workouts without any intervention from the research staff. Since beach handball tournaments usually encompass 2 daily matches, the training regimen during the training camps encompassed 1 daily morning session mainly focused on sand-based physical conditioning and individual technical skills and 1 daily afternoon session mainly focused on team tactical trainings and small-sided games. All training sessions lasted ~1.5 h and they were composed by ~15 min of warm-up without and with balls, ~1 h of specific work, and ~15 min of cool-down and stretching exercises.

### 2.3. Procedures

During each experimental session, the workload was objectively recorded by means of HR monitors (H7, Polar Team System, Kempele, Finland). The duration of each training session was recorded to successively recognize the HR corresponding to the training activities. For matches, the entire playing time was considered. The 30 min of standardized warm-up preceding each match and the between-halves rest times were excluded from the analysis. After each session, the HR data were exported in 1 s epochs via proprietary software and the individual workload was calculated according to the SHRZ method [14]. This methodology allowed us to identify the individual workload score by calculating the product of the accumulated session duration (min) of 5 HR zones by a coefficient relative to each zone (50–59.9% of HRmax = 1, 60–69.9% of HRmax = 2, 70–79.9% of HRmax = 3, 80–89.9% of HRmax = 4, 90–100% of HRmax = 5). Then, the SHRZ workload (in AU) was calculated by summating the results. According to previous methodology used in sand-based sports [22,23] and other team sports [24], the peak HR registered across training sessions and matches was considered for the calculation of the SHRZ workload [24]. Data were subsequently expressed as percentages of the HRpeak.

Furthermore, the workload was subjectively assessed by means of the sRPE method [17,25]. Since recent evidence has suggested that RPE scales are interchangeable [26,27], in the present study, the category-ratio 10 (CR10) scale modified by Foster et al. [25] was administered by asking each player: “How hard was your training/match?” within 30 min after the completion of each training session and each match. The sRPE workload was then calculated by multiplying the individual score of the CR10 scale for the duration (min) of the training/match [25].

### 2.4. Preliminary Analysis

Means and standard deviations were calculated for each analyzed variable. Normal distribution was verified by the Shapiro–Wilk test. The Shapiro–Wilk test showed that the sRPE and SHRZ were not normally distributed when all of the sessions were combined. However, the sRPE and SHRZ showed different distribution patterns when training and matches were split. These results highlight that, in team sports, data could vary between subjects and sessions. Thus, the intersubject variability should be considered when analyzing data in order to avoid inaccurate results emerging from an over- or under-estimation of statistical significance in repeated measures of the study design [28].

### 2.5. Statistical Analysis

The overall relationship between the SHRZ and sRPE methods was assessed by means of the Pearson product moment and Spearman correlations, and then with linear regression. The sample was analyzed by combining all of the sessions and subsequently dividing trainings and matches. The magnitude of correlations was defined by the following criteria: trivial (<0.1), small (from 0.1 to 0.29), moderate (from 0.3 to 0.49), large (from 0.5 to 0.69), very large (from 0.7 to 0.89), and almost perfect (from ≥0.9 to 1) [29,30]. Additionally, the relationships between the SHRZ and sRPE methods were analyzed via LMM using the sRPE and SHRZ values as fixed effects while the random effects were represented by the individual response of each player. First, the models were fitted with only random intercepts for each player. However, by merely fitting the random intercept at the subject level, the variability of each player between sessions was not taken into consideration. Therefore, subsequently random slopes of the relationship between the SHRZ and sRPE were fitted into the models. Bryk/Raudenbush R-squared (R^2^) values were calculated for each random intercepts LMM. Finally, the likelihood-ratio test was used to compare the each LMM developed with the linear regression analysis and to compare the 2 LMMs with only random intercepts, and with random intercepts and random slopes. Statistical analysis was performed using STATA statistical software version 15.1 (StataCorp, College Station, TX, USA) and the level of significance was set at *p* < 0.05.

## 3. Results

Descriptive characteristics of players are presented in Table 1.

**Figure 1:**
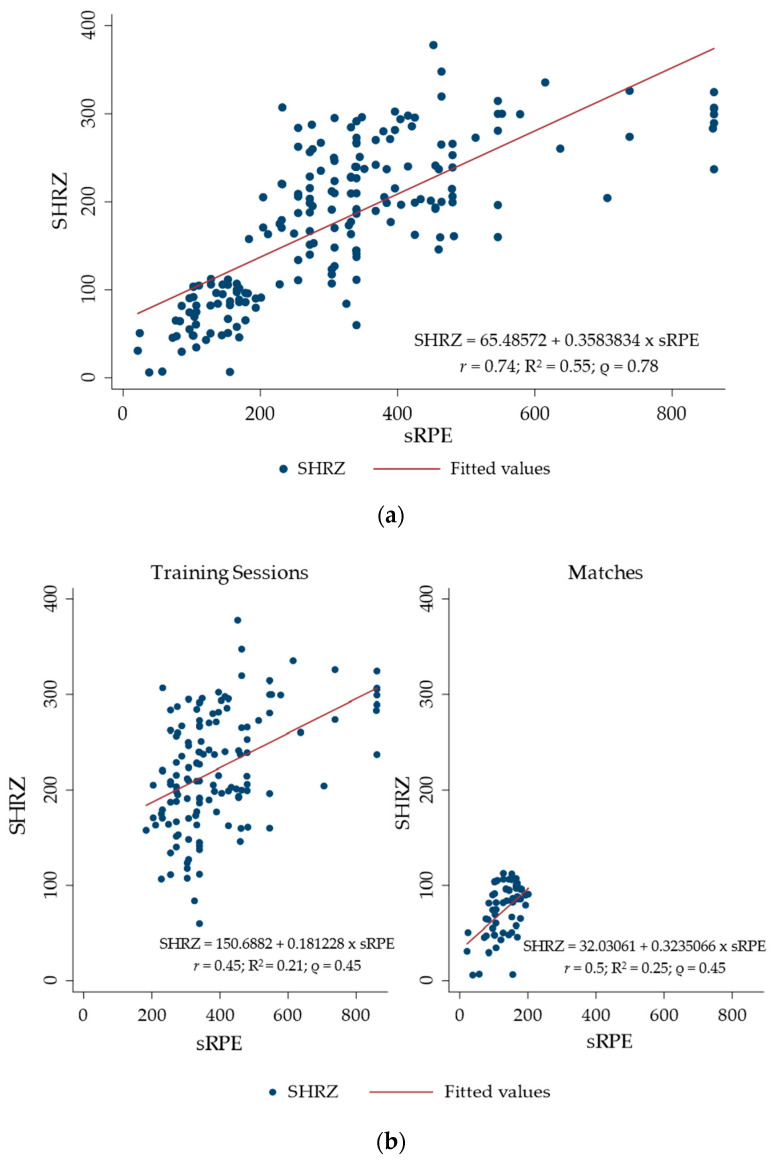
Relationship between the Summated Heart Rate Zone (SHRZ) (y axis) and session Rating of Perceived Exertion (sRPE) (x axis) for all sessions (**a**) and for training sessions and matches separately (**b**).

When combining training sessions and matches, results revealed a %HRpeak of 71.3 ± 8 (training sessions: 70.1 ± 6.5 %HRpeak; matches: 74.2 ± 10.5 %HRpeak), a SHRZ workload of 178.8 ± 13.2 AU (training sessions: 222 ± 61.0 AU; matches: 73.2 ± 27.9 AU), and a sRPE workload of 315.4 ± 178.2 AU (training sessions: 392.9 ± 153.1 AU; matches: 127.1 ± 42.8 AU). The correlation coefficients for the overall relationship between the SHRZ and sRPE methods were very large (*r* = 0.74; R^2^ = 0.55; ρ = 0.78) when combined training sessions and matches were assessed. When training sessions were studied singularly, moderate (*r* = 0.45; R^2^ = 0.21; ρ = 0.45) correlation coefficients were shown. When only matches were considered, moderate-to-large (*r* = 0.5; R^2^ = 0.25; ρ = 0.45) correlation coefficients were shown. Relationships investigated via linear regression are graphically shown in Figure 1.

The first fitted LMM included random intercepts for each player by adding a random-effects part on the linear regression model for the whole sessions. The estimated standard deviation (SD) of the random intercepts was 28.2 AU (95% confidence interval: 16.8–47.3), with a standard error of 7.4 and R^2^ = 0.61. The likelihood-ratio test showed that this model offered significant (Chi^2^: 25.2; *p* < 0.001) improvement over a linear regression model with only fixed effects, meaning that the intercepts were significantly different between players. When applying the same procedure exclusively to training sessions, the SD of the estimated random intercepts was 34.3 AU (95% confidence interval: 21.8–53.9), with a standard error of 7.4 (R^2^ = 0.33). Similarly, the likelihood-ratio test proved that this model was significantly (Chi^2^: 41.8; *p* < 0.001) better than the linear regression model with only fixed effects. Considering only the matches sessions, the SD of the estimated random intercepts was 15.6 AU (95% confidence interval: 8.8–27.4), with a standard error of 4.5 and R^2^ = 0.39. Likewise, the likelihood-ratio test proved that this model was significantly (Chi^2^: 12.8; *p* < 0.001) better than the linear model with only fixed effects.

Overall, including random slopes into the developed models did not bring significant improvements with respect to the random-only intercepts LMMs when training sessions and matches were separated (*p* > 0.05). However, when considering all of the sessions together, the developed model showed significant (*p* < 0.001) player-to-player variation in the slope coefficients, with a significant improvement (*p* < 0.05) with respect to the only random intercepts model (Table 2).

**Figure 2:**
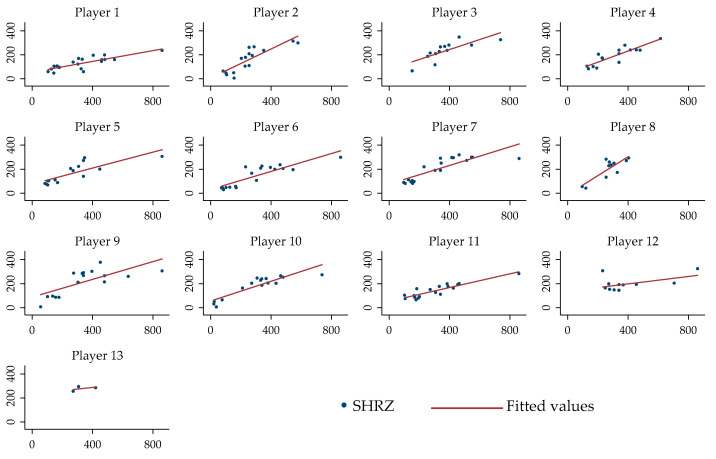
Relationship between the SHRZ (y axis) and sRPE for the individual players’ responses for all the sessions.

Visual representation of the relationships between the SHRZ and sRPE with different intercepts and slopes across each player for the whole sessions are displayed in Figure 2.

Random intercepts and slope coefficients for each player based on the whole session LMM are reported in Table 3.

To clarify the relationship between the SHRZ and sRPE, Equation (1), combining the fixed and random slopes sRPE, was developed:

SHRZ = 61.19 + (u1j + 0.39) sRPEij + U0j+ єi(1)

**Figure 3:**
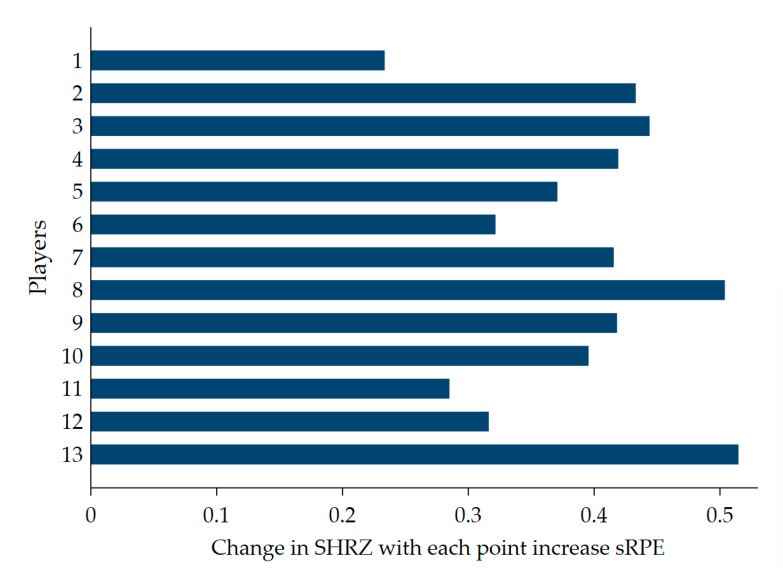
Interindividual variability of the relationship between the sRPE and SHRZ workloads. In some players (for example, 13 and 8), the SHRZ increased most steeply as the sRPE increased, whereas, in player 1, the increase was about the half of the abovementioned players.

In other words, the slope for player equals the fixed-effect slope for the whole sample plus the random-effect slope for that player. Figure 3 displays the calculated 13 combined slopes for each player. For player number 8, for instance, the combined slope was u1j (+ 0.11 for player 8) + 0.39 = 0.5.

## 4. Discussion

The present study aimed to assess the correlation between objective and subjective measures of internal load in team sports, such as beach handball, and to investigate this relationship by considering the individuality of players by means of LMM. Results showed that LMM can give more powerful and appropriate information regarding the relationship between SHRZ and sRPE workloads rather than the usual procedure using correlations and linear regression with only fixed effects.

In line with studies investigating the indoor handball characteristics [8,31,32,33], many aspects of beach handball, such as physiological parameters [22,34], individual and team performance [35,36], and shooting actions [37,38,39], have been investigated. However, no previous study has investigated the relationship between the objective (SHRZ) and subjective (sRPE) methods used for assessing the players’ internal load.

Our results showed a very large relationship between the SHRZ and sRPE methods, independently from the type of session. When looking at training sessions and matches separately, this relationship was moderate and moderate-to-large, respectively. The trend was confirmed by the results of the linear regression analysis, showing a large relationship when the sessions were analyzed as a whole and small relationships when the training sessions and matches were analyzed separately. For other team sports, correlation coefficients have shown a strong [40], high [41], or very high relationship [42], promoting the sRPE as a useful method for monitoring internal load in youth trainings. However, in the case of team sports, not only the team as a whole has to be considered, but also the interindividual variability when analyzing workload data. The response to exercise training may not only differ between athlete, but also within the same athlete on different sessions. Previous studies have indicated that correlation coefficients for the relationship between internal load assessed using HR-based methods and via sRPE ranged between *r* = 0.71 for soccer [42] and *r* = 0.85 for basketball [40] when the team was analyzed as a whole. When within-athlete correlation coefficients were calculated, values ranged between *r* = 0.8 and *r* = 0.96 for basketball [40], *r* = 0.5 and *r* = 0.77 for soccer [42], and *r* = 0.62 and *r* = 0.93 for beach volleyball [43]. However, when multiple players are monitored across multiple sessions, the tendency to summarize the data with a single number may lead to the exclusion of intra- and intersubject variability from the analysis [19]. In fact, for team sports, models based on physiological parameters might underestimate the internal load during anaerobic and high-intensity activities, underlying the higher sensitivity of the sRPE method to workload changes, especially during the transition from base to higher intensities of conditioning programs [44]. Thus, simply measuring the strength of a relationship using correlations, without taking into account changes in an individual predictor variable, may lead to a misinterpretation of the relationship between two variables [19]. Furthermore, one of the most common issues occurring during data collection is represented by missing data [45]. For this reason, LMMs should be used, since they have the advantage to handle missing data without removing participants from the analysis [46].

This study aimed to analyze the relationship between SHRZ and sRPE by means of LMM. For the analysis, only random intercepts were initially used. However, the SHRZ workload increased as the sRPE workload increased (Figure 1), with different individual responses (Figure 2). To overcome the issue of interindividual variability, it was hypothesized that adding random slopes to the model would help to deeply investigate the relationship. Currently, the use of mixed models is becoming popular among sport science research. Govus et al. [47] used the LMM to analyze the relationship between subjective wellness score and external load, between external load and sRPE, and between subjective wellness score and sRPE in American college football players. For the LMM, the authors used the random intercept for athletes (to calculate the intraindividual variability) and the random slope for training sessions (to model a separate slope for the different types of training sessions). LMMs have also been used to evaluate the effects of individual characteristics (i.e., playing position, playing time, or playing experience) and contextual factors (i.e., season phases, previous game outcome, or opponent level) on three dependent variables (weekly training load, pre-game recovery, and performance index rating) in basketball [48] and to investigate [49] workload and well-being across games played on consecutive days during the in-season phase in basketball players, with the game day as the fixed effect and players, opposition rank, location, and score difference as random effects. It is therefore evident that LMMs are more commonly applied when analyzing data of relative workload in team sports.

Although this study provides interesting insights for coaches and sport scientists, some limitations should be acknowledged. First, the sample encompasses only youth beach handball male players. Therefore, future research should be carried out to investigate any potential difference in the internal load in players of different ages and/or gender. Moreover, the use of LMMs is becoming more common when analyzing team sports data, for example, to assess the relationships between external and internal load [47] or between workload and well-being data [49]. However, no previous study has used LMMs to correlate subjective and objective measures of internal load. Thus, no comparisons were allowed, and it should be verified whether the proposed statistical model could also be meaningful in other team sports.

## 5. Conclusions

The main findings suggest that subjective perception of internal load experienced by youth beach handball players increases with the objective internal load. However, the increase varies between players and sessions. To correlate those two measures to monitor the internal load, simple correlation is usually performed. However, correlation does not allow for the consideration of the intra- and interindividual variability which occurs when working with team sports, and it is not possible to handle missing data, resulting in a loss of information. To overcome with these issues, LMMs represent a more appropriate and powerful statistical approach for providing a more comprehensive view of the players’ responses to a given training stimulus. Therefore, researchers are encouraged to apply LMMs rather than simple correlations to analyze internal load.

## Figures and Tables

**Table 1:** Players’ descriptive characteristics. Values represent mean ± standard deviation (SD).

Characteristics	Mean ± SD	[95% CI]
Age (years)	15.9 ± 0.3	15.8–16.1
Weight (kg)	67.4 ± 6.8	62.2–72.7
Height (m)	1.8 ± 0.1	1.8–1.9
BMI (kg·m^−2^)	20.4 ± 1.5	19.2–21.6
Heart Rate Peak (beat∙min^−1^)	195.9 ± 8	191–200.7

Note: CI: Confidence Interval; BMI: Body Mass Index; Heart Rate peak: Peak heart rate registered across training sessions and matches.

**Table 2:** Comparison of Linear Mixed Models developed for the whole sessions.

	Coef.	SE	z	*p* > |z|	[95% CI]	
(A) Random Intercept Model						
sRPE-SHRZ Relationship	0.36	0.02	16.82	0	0.32	0.40
Intercept	70.92	11.05	6.42	0	49.26	92.58
					*p* < 0.001	
(B) Random Intercept plus Random Slope Model						
sRPE-SHRZ Relationship	0.39	0.03	11.98	0	0.33	0.45
Intercept	61.19	7.65	7.99	0	46.18	76.19
					*p* < 0.001	
Likelihood-Ratio test (Model A vs. Model B): *p* < 0.05						

Note: sRPE: Session Rating of Perceived Exertion; SHRZ: Summated Heart Rate Zone; coef.: Coefficient; SE: Standard errors; CI: Confidence Interval.

**Table 3:** Random intercepts and random slope coefficients for each player based on the whole session Linear Mixed Model.

Player	Random Slope (Mean)	Random Intercept (Mean)
1	−0.16	−2.02
2	0.04	−2.23
3	0.05	1.02
4	0.03	−0.17
5	−0.02	0.88
6	−0.07	−2.91
7	0.03	1.30
8	0.11	0.61
9	0.03	1.81
10	0.01	−0.30
11	−0.11	−1.64
12	−0.07	1.63
13	0.12	2.01

## Data Availability

The data presented in this study are available on request from the corresponding author.
